# Mosquito magnet^®^ liberty plus trap baited with octenol confirmed best candidate for *Anopheles* surveillance and proved promising in predicting risk of malaria transmission in French Guiana

**DOI:** 10.1186/1475-2875-13-384

**Published:** 2014-09-26

**Authors:** Samuel B Vezenegho, Antoine Adde, Pascal Gaborit, Romuald Carinci, Jean Issaly, Vincent Pommier de Santi, Isabelle Dusfour, Sébastien Briolant, Romain Girod

**Affiliations:** Medical Entomology Unit, Institut Pasteur de la Guyane, 23 Avenue Pasteur, BP 6010, 97306 Cayenne Cedex, French Guiana; Direction Interarmées du Service de Santé en Guyane, Quartier La Madeleine, BP 6019, 97306 Cayenne Cedex, French Guiana; Laboratory of Parasitology, Institut Pasteur de la Guyane, 23 Avenue Pasteur, BP 6010, 97306 Cayenne Cedex, French Guiana; Epidémiologie des parasitoses tropicales (EPaT) Team (EA 3593), UFR de Médecine, Université des Antilles et de la Guyane, Campus Saint-Denis, Avenue d’Estrées, 97306 Cayenne cedex, French Guiana; Institut de Recherche Biomédicale des Armées, BP 73, 91223 Brétigny sur Orge cedex, France

**Keywords:** *Anopheles darlingi*, Mosquito Magnet^®^ trap, Human landing catches, Entomological surveillance, Malaria, French Guiana

## Abstract

**Background:**

In French Guiana, Mosquito Magnet^®^ Liberty Plus trap baited with octenol (MMoct) has been proposed for sampling *Anopheles darlingi* after comparison with CDC light trap and Human landing catch (HLC). However, other available lures were not tested. The current study compared MMoct and MM baited with Lurex™ (MMlur) to HLC, and analysed entomological data from MMoct collection with malaria cases to facilitate malaria surveillance.

**Methods:**

Two independent experiments were conducted during 2012 and 2013 in Saint-Georges town, French Guiana. The first experiment used Latin square design to compare MMoct and MMlur to HLC between 18:30 to 22:30 and 05:00 to 07:00. Parity rate was determined for *An. darlingi* from each sampling system. In the second experiment, a 24:00 hour collection was done for four consecutive days during the first week of each month and every four days for the rest of the month using MMoct. Portion of the 24 hour collection was dissected for parity rate. All anophelines were screened for *Plasmodium* infection by PCR. Data for number of malaria cases was analysed for association with density of *An. darlingi*.

**Results:**

In the first experiment, 3,721 anopheline mosquitoes were collected over 21 nights. Of these, 95.7% was identified morphologically to five species and *An. darlingi* contributed 98.4%, mainly from HLC (75.1%, CI 95% [73.2-77.0]) than MMoct (14.1%, CI 95% [12.6-15.7]) and MMlur (10.8%, CI 95% [9.4-12.2]). Species richness was highest in HLC meanwhile species diversity index was greatest in MMoct. MMoct collected more parous *An. darlingi* than HLC (p < 0.0001) and MMlur (p = 0.0021). The second experiment amounted to 2035 females, 60.8% belonging to 10 species. *Anopheles darlingi* constituted 85.0% of the species and had parity rate of 52.3%. Specimens were uninfected with *Plasmodium*. Density of *An. darlingi* best correlated with malaria cases observed six weeks later (p = 0.0016; r = 0.4774).

**Conclusion:**

Though MMoct and MMlur performed well in sampling *An. darlingi*, MMoct captured more species and, therefore, would be useful for surveillance. Even if it collected mostly parous mosquitoes, MMoct proved useful in collecting entomological data required for predicting malaria emergence. It is a potential replacement for HLC.

## Background

Although malaria clinical cases have dropped significantly since 2005, the disease remains a major public health problem in French Guiana, an overseas territory of France located in the north-east of South-America. Recent data indicates that *Plasmodium vivax* is currently responsible for majority of malaria cases in the country. The disease is now concentrated in the Eastern region, meanwhile the coastal region, where approximately 75% of population lives, experiences primarily imported cases even though autochthonous transmission is frequently observed [1].

Malaria transmission in French Guiana is maintained by *Anopheles darlingi*, a species belonging to the subgenus *Nyssorhynchus*. Incrimination of *An. darlingi* is attributed to its predominantly anthropophilic behaviour, occurrence in high density, wide distribution and susceptibility to infection by *Plasmodium* sporozoites resulting in high prevalence of infected females [2–5]. Besides *An. darlingi,* recently other species: *Anopheles nuneztovari sensu lato* (*s.l.*)*, Anopheles oswaldoi s.l.* and *Anopheles intermedius* have been shown to occur in high numbers in the country and found naturally infected with *Plasmodium* sporozoites [6]. However, their role in malaria transmission in French Guiana is largely unknown.

In a context of the emergence of a new political will to eliminate malaria all over the territory, there is a need to set up entomological surveillance systems to accurately measure malaria transmission and set priorities for the control of malaria vectors in French Guiana. Current control strategies in French Guiana are mainly through indoor spraying of residual insecticides and distribution of long-lasting insecticide-treated bed nets. Both approaches aim to reduce both vector density and contact between human and malaria vectors at a level likely to disrupt malaria transmission [7].

Surveillance systems are critical if reliable entomological data are to be used for assessing malaria transmission. The human biting rate and the entomological inoculation rate (EIR) are key factors used to measure malaria transmission. These are widely calculated from host seeking female mosquitoes collected by the gold standard human landing catch (HLC) [8]. HLC directly measures biting rate of anthropophagic mosquitoes involved in transmission [9] but suffers serious criticism. This include ethical concern over the risk of exposure of collectors to infective bites of mosquitoes, issues of efficiency and reproducibility in relation with the variability in human attractiveness, questions of labour intensiveness, requiring employment of many collectors and their strict supervision [10, 11]. These disadvantages resulted in development of a number of alternative mosquito trapping methods over the past decade, especially light traps such as the CDC miniature light trap [12], double net traps with Mbita trap as an example [13] and odour baited traps such as the Mosquito Magnet^®^ traps (Woodstream Corporation Lititz PA). The efficacy of these trapping methods in measuring vector density varies and depends largely on the behaviour of individual mosquito species [14]. Limited studies in French Guiana have compared sampling systems for anopheline mosquitoes. The most recent is by Dusfour *et al.* [15] who compared the efficiency of Mosquito Magnet^®^ traps baited with octenol (MMoct) and CDC-light traps to HLC for sampling anopheline mosquitoes in Camopi, a forested malaria area. The conclusion was that, MMoct is an alternative method to HLC. Prior to the current study, the efficiency of octenol compared to Lurex™, an available lure used in MM has never been tested in French Guiana.

In endemic inland villages of French Guiana, anopheline mosquitoes are considered to rest on vegetation (outdoors) as majority of human living structures have roof and no walls. In line with health authorities’ objectives of maintaining the current gains in decreasing malaria, the choice of an outdoor sampling tool for surveying malaria transmission and guide vector control operations becomes of paramount importance.

In this context, the present study aims to evaluate MM using two separate lures (octenol and Lurex™) against HLC based on density, diversity and parity of anopheline mosquitoes, and to test the best system after evaluating sampling techniques to collect entomological data which together with clinical malaria cases can facilitate malaria surveillance. Lurex™ which is readily available and cost effective was tested for the first time in French Guiana.

## Methods

### Study site

Experiments were conducted in the Saint-Georges town, located along the Oyapock River separating French Guiana from Brazil. The study area is inhabited by around 2,000 people being essentially Creoles or Amerindians, including people who originated from Brazil. The climate is tropical characterized by four seasons: a long rainy season (from April to June), a long dry season (from July to December), a short rainy season (from January to February) and a short dry season in March. Average temperature is about 27°C with average relative humidity of 80%.

**Figure 1:**
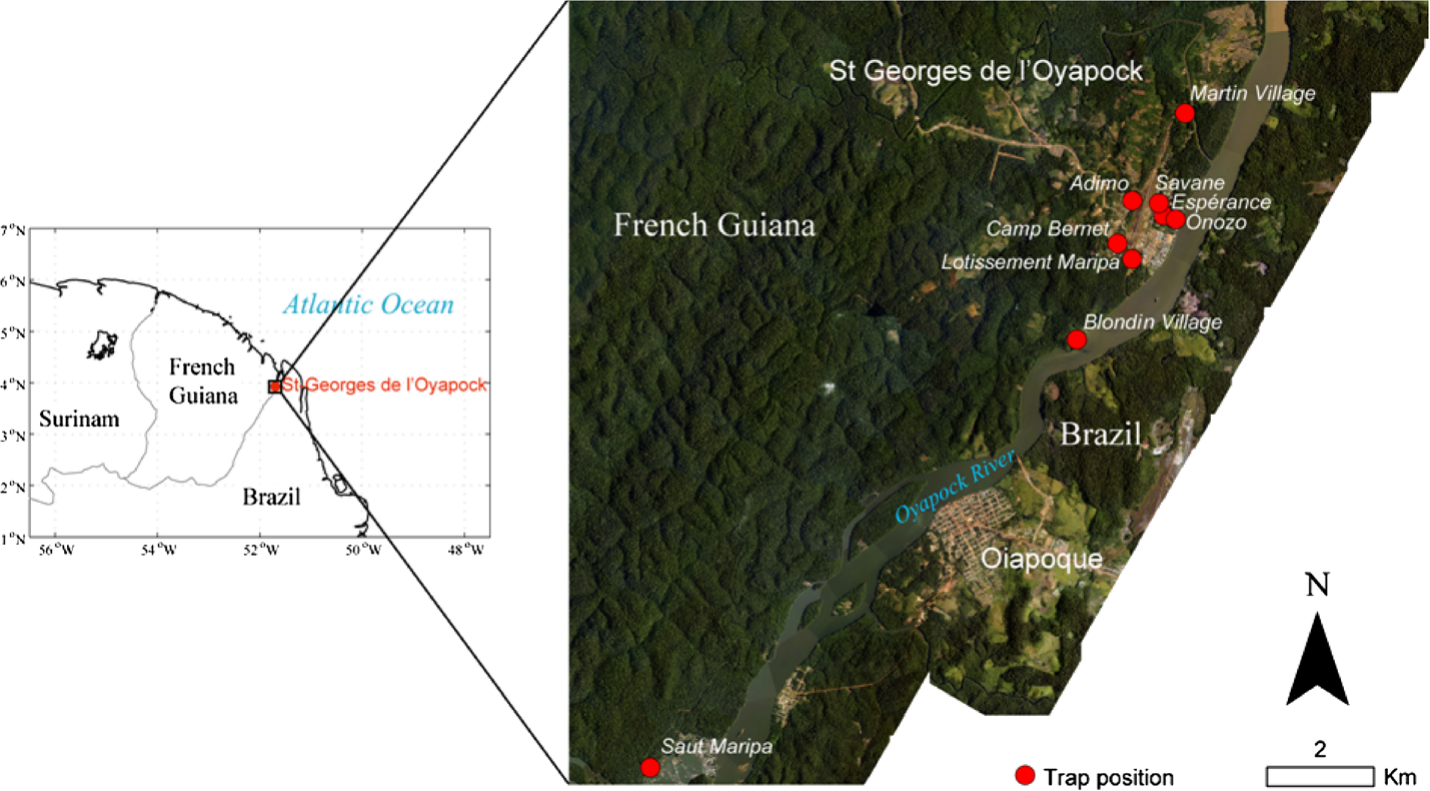
**Aerial photograph (spatial resolution of 50 cm) of the town of Saint-Georges obtained from the French National Geographic Institute (BD-ORTHO** ^**®**^ **product) in February 2014.**

Location and description of mosquito collection sites are summarized in Figure 1 and Table 1 respectively. Five of the areas (Onozo, the military base at Camp Bernet, Blondin village, Saut Maripa and Martin village) are located along the Oyapock River or its tributaries. The other areas, Espérance, Adimo, Lotissement Maripa and Savane are located inland. Collection areas were chosen for their high mosquito density and history of malaria cases. Houses near the river are mostly constructed of wooden walls with corrugated aluminium roofs without ceilings and are suspended approximately a meter above the ground surface. However, housing type inland of the town is predominantly brick walled with corrugated aluminium sheets as roof.

**Table 1:** **Description of areas in the region of Saint-Georges used for mosquito sampling using MMoct**

Characterization of sampling area			
Name of area	Coordinates	Access	Environment
Adimo	3°53′ 55,8″N; 51°45′ 21,2″O	Road	Savannah
Savane	3°53′ 54,2″N; 51°48′ 5″O	Road	Savannah
Espérance	3°53′ 46,7″N; 51°48′ 2,1″O	Road	Savannah
Lotissement Maripa	3°53′ 29,7″N; 51°48′ 30″O	Road	Savannah
Onozo	3°53′ 44,5″N; 51°47′ 54,8″O	Road	Savannah
Camp Bernet	3°53′ 20″N; 51°48′ 21,2″O	Road/water	Savannah/Forest
Saut Maripa	3°48′ 10,9″N; 51°53′ 12,9″O	water	Forest
Martin village	3°54′ 49,2″N; 51°47′ 49″O	water	Forest
Blondin village	3°52′ 31,2″N; 51°53′ 12,9″O	water	Forest

Saint-Georges town was chosen for this study due to its accessibility by road from Cayenne, the main town in French Guiana where the medical entomology unit of the Pasteur institute is based and especially because the dynamics of malaria is well-documented. Indeed, there is a health centre where malaria cases have been diagnosed and treated for several years. The epidemiological profile in the study area is well known, characterized by a single peak of malaria cases between October and December largely due to *P. vivax.* However, malaria cases due to relapse are observed months after peak in transmission.

### Sampling methods used

Two sampling techniques consisting of MM and HLC were used in this study. Mosquito Magnet trap was originally developed for residential use and the lures used are not specific to species of anopheline mosquitoes. To compare the attractiveness of mosquitoes to available lures, MM was fitted with a cartridge containing either the lure octenol (MMoct) or Lurex™ (MMlur) which were replaced after 15 days. MM trap operate on a counterflow technology to lure and capture mosquitoes. Propane gas from a tank is catalytically converted into carbon dioxide (flow rate of 500 cc/min), heat and moisture. These products create a plume of odour cue exhausted outside the trap through a central pipe by a small fan and entice female mosquitoes to follow the plume to its source [16]. The central pipe is surrounded by a wider tube through which a larger fan sucks air upward, carrying along attracted mosquitoes into nylon net in the trap. Human landing catches were performed strictly according to WHO recommendation [17]. In brief, this is performed by exposing the lower legs and collecting landing mosquitoes with a mouth aspirator.

### Study design and mosquito collection

This study was conducted from September 2012 to February 2013 (six months) then from September to November 2013 (three months). Two independent experiments were carried out during each period.

The first experiment was conducted in Blondin village. MMoct and MMlur traps were evaluated against HLC in a 3 × 3 Latin square design replicated three times during each periods. The collection sites were located at least 50 m apart. Traps and collection by HLC were rotated randomly through the collection sites, over three consecutive days. In the course of this experiment, two individuals each acting as both bait and collector performed HLC. Mosquito collections using MMoct, MMlur and HLC were done from 18:30 to 22:30 and from 05:00 to 07:00. The hours of collection were chosen to cover the period of maximum aggressiveness of anopheline mosquitoes in French Guiana.

In a second experiment performed in Saint-Georges town, only MMoct was used to follow up entomological parameters. For each study period seven areas were used, each with a trap located next to a house in which people live. A description of the collection areas is provided in Table 1. Mosquitoes were collected from Martin village, Camp Bernet, Lotissement Maripa, Adimo, Espérance, Onozo and Savane during the first period and during the second period, two of the areas: Onozo and Espérance were replaced by Blondin village and Saut Maripa. Mosquitoes were recovered from the traps after 24 hours for four consecutive days during the first week of the month. For the rest of the month, mosquitoes were recovered every Monday and Thursday at 08:00 by personnel from a contracted association established in Saint-Georges.

### Mosquito processing

Mosquitoes collected were transported to the health centre and sacrificed by freezing at -20°C for 30 minutes. Specimens were then sorted, counted and identified morphologically using taxonomic keys specific for the region [3, 18, 19]. A subset, consisting of at most 10 *An. darlingi* caught during sampling sessions in the first experiment, as well as those collected during the first week in the second experiment were dissected and parity status determined by examining the coiling or uncoiling of ovarian tracheoles [20]. Mosquito with dilated tracheoles at the distal end was termed parous. Those with coiled tracheoles and not dilated at the distal end were labelled nulliparous. Parous mosquitoes have laid eggs at least once while nulliparous mosquitoes have never laid eggs. Parity rate is used to determine the age structure of mosquito population and linked to their role in malaria transmission. Collections made during the rest of the weeks in the second experiment were stored for long and were too dry to be dissected. All specimens were then individually stored in 1.5 mL Eppendorf tubes containing silica gel, provided with a unique code before being transported to the Institut Pasteur in Cayenne for further processing. Once at the laboratory, DNA was extracted from the head and thorax of all anophelines female collected in the second experiment. Extraction was according to the protocol provided by the QIAgen DNeasy Blood and Tissue kit (QIAgen Ltd., Crawley, UK). The resultant DNA was subjected to polymerase chain reaction (PCR) according to Snounou *et al*. [21] to identify *Plasmodium* sporozoite infection.

### Epidemiological data

Malaria diagnosis was routinely carried out at the local health post in Saint-Georges town using SD Bioline Ag pf/pan (Standard Diagnostics Inc) kit, a rapid diagnostic test. An anonymous list of clinical malaria episodes for people who lived and/or contracted malaria in areas of the town of Saint-Georges where mosquito sampling was carried out during the study periods, was provided by the regional office of national institute for public health surveillance. The list consisted of date of first clinical signs, the usual place of residence and/or the presumed place of contamination if the patient declared to have spent at least one night outside his home, 10–20 days before the appearance of clinical signs. Patients with more than one episode of malaria within three weeks were counted as one case of infection and if more than three weeks, this was considered to be two separate cases.

### Data analysis

For the first experiment, statistical analysis was performed with lme4 package in software R (version 3.0.2.). A generalized linear mixed model was used to compare different sampling systems based on the total number of anopheline mosquitoes caught per six hours in a night during all the study period. This model test the effect of a fixed independent variable (sampling system) while controlling random effect (location of the sampling system) and taking into account repeated measures. Parous rates were compared by Fisher’s exact test in GraphPad Prism version 5 (GraphPad Software Inc.). Anopheline species diversity index was calculated according to Simpson [22]. This index takes into account the number of species present (richness) and a measure of the relative abundance of the different species (evenness). Data from the second experiment were first aggregated on a weekly base. A zero to eight weeks lag time between densities of adult female *An. darlingi* collected in MMoct and clinical malaria cases was analysed for correlation using non-parametric Spearman’s test.

### Ethical concerns

Female mosquitoes were collected using HLC with the aid of mouth aspirators when landing on collector legs. Collectors were local volunteering residents. They were provided training on HLC and informed of the associated risks of the collection method and were supervised during the captures by the authors. Malaria prophylaxis was proposed and information on the medication was provided. Collectors who benefited from prophylaxis gave their free, express and informed consent. The number of weekly malaria cases used for the study was provided by the regional office of national institute for public health surveillance.

## Results

### First experiment

During 21 nights of evaluating sampling methods in Blondin village, 3,721 anopheline mosquitoes were collected and 95.7% (n = 3,562) of these were identified into five species (Table 2). *Anopheles darlingi* was the predominant species contributing 98.4% of the identified specimens. This was followed by *An. nuneztovari* (0.9%), *Anopheles triannulatus* (0.6%), *Anopheles braziliensis* (0.1%) and *Anopheles mediopunctatus* (0.1%).

**Table 2:** **Anopheline mosquitoes collected during experiment one from Blondin village using two variants of MM traps and HLC performed by two collectors**

	Sampling method		
Species (Total)	% MMLur [CI95]	% MMoct [CI95]	% HLC* [CI95]
*An. darlingi* **(2 006.5)**	10.77 [9.4-12.2]	14.10 [12.6-15.7]	75.13 [73.2-77.0]
*An. triannulatus* **(12.5)**	0	80.00 [48.7-96.5]	20 [3.5-51.3]
*An. nuneztovari* **(18.5)**	0	48.65 [25.2-72.5]	51.35 [27.5-74.8]
*An. mediopunctatus* **(1.0)**	0	0	100.0 **[2.5]**
*An. braziliensis* **(1.5)**	0	0	100 **[8.6]**
**Total (2 040)**	**10.6 [9.3-12.0]**	**14.80 [13.3-16.4]**	**74.61 [72.7-76.5]**

*Data for HLC is shown as number of mosquitoes collected per human. % is mean percentage; CI95, Confidence interval at 95%.

Collections made using HLC constituted 74.6% (CI 95% [72.7%-76.5%]) of the known anopheline species collected whereas MMoct and MMlur captured 14.8% (CI 95% [13.3%-16.4%]) and 10.6% (CI 95% [9.3%-12.0%]) respectively. Of the anopheline mosquitoes caught using HLC and MMoct, 99.0% (CI 95% [98.4%-99.5%]) and 93.7% (CI 95% [90.3%-96.2%]), were, respectively, *An. darlingi*. MMlur collected only *An. darlingi*.

Results of statistical analysis using generalized linear mixed model to compare the total number of mosquitoes caught by each sampling system is presented in Table 3. It showed statistical significant difference in anopheline mosquito density between the sampling systems (p < 0.05). Similar results were obtained when number of *An. darlingi* sampled using HLC was compared with those of either MMoct or MMlur. However, MMoct and MMlur were not statistically different (p > 0.05) in sampling this species. Species richness was highest for HLC (five species), followed by MMoct (three species) and MMlur (one species). On the other hand, Simpson’s diversity index was highest for MMoct (0.12) followed by HLC (0.02) and MMlur showed no species diversity. The ratio of *An. darlingi* captured by MMoct and MMlur with reference to HLC as standard were respectively 0.05 and 0.11 according to the generalized linear mixed model. In contrast, MMoct collected more *An. triannulatus* than HLC*.* It captured four times the number of *An. triannulatus* collected by HLC. HLC and MMoct had equal efficiencies in sampling *An. nuneztovari*.

**Table 3:** **Statistical difference in number of anopheline mosquitoes trapped by sampling systems compared**

Response variable	Sampling systems	Estimate	SE	t-value	p-value
*Anopheles*	MMlur *vs*. HLC	1,9685	0,5098	3,861	0,000974
	MMoct *vs*. HLC	2,9796	0,4737	6,291	0,000004
	HLC vs. MMlur	0,26314	0,06264	4,201	0,00044
	MMoct vs. MMlur	-0,63737	0,26607	-2,396	0,026471
*Anopheles darlingi*	MMlur *vs*. HLC	2,1845	0,6109	3,576	0,001891
	MMoct *vs*. HLC	3,0765	0,5281	5,825	0,000011
	HLC vs. MMlur	0,2113	0,0561	3,767	0,001212
	MMoct vs. MMlur	-0,4763	0,2539	-1,876	0,075328

Mean catch +/- SE associated with the generalized mixed linear model for the number of mosquitoes sampled for the sampling systems compared. HLC: Human landing catch, MMlur: Mosquito Magnet baited with Lurex™, MMoct: Mosquito Magnet baited with octenol, Estimate difference in least squares means, SE: Standard error.

The parity rate of *An. darlingi* collected by sampling methods evaluated was determined during this study by dissecting a total of 496 *An. darlingi* (Table 4). The proportion of parous females collected using MMoct was significantly higher than that collected using HLC (p < 0.0001) and MMlur (p = 0.0021). However, no difference was obtained between proportions of parous females collected using HLC and MMlur (p > 0.05).

**Table 4:** **Percentages of parity for** ***Anopheles darlingi*** **collected in Blondin village by three sampling methods**

Sampling method	Number dissected	Indeterminate	MPR	CI 95%
HLC	320	5	**45.1**	**39.50-50.8**
MMlur	50	2	**45.8**	**31.3-60.8**
MMoct	126	3	**56.9**	**47.5-65.8**

MPR: Mean percentage of parity rate. CI 95%, Confident interval at 95%.

### Second experiment

Results of longitudinal sampling done from nine localities in the town of Saint-Georges are presented in Table 5. A total of 2,035 anopheline females were collected and the majority (35.6%) was from Blondin village. In all, 60.8% (n = 1,237) of the global anopheline mosquitoes were identified as belonging to 10 different species (Table 5). *Anopheles darlingi* constituted 85.0% (n = 1,052) of the identified species. The three other abundant species were *An. braziliensis* (5.8%), *An. triannulatus* (5.3%) and *An. nuneztovari* (2.9%). The bulk of *An. darlingi* (52.1%, n = 548) was collected from Blondin village and the least collection (0.4%, n = 4) was from Savane. *Anopheles darlingi* had a parity rate of 52.3% (n = 91)*.* Parity for five of the dissected specimen could not be determined. None of the mosquitoes collected in the second experiment was positive for *Plasmodium* infection.

**Table 5:** ***Anopheles*** **species composition from the nine localities in the town of Saint- Georges collected using MMoct**

Collection area	Species	Number	Species richness	Simpson diversity index	Anophelines percentage
Adimo	*An. braziliensis*	34	4	0.55	4.4
	*An. darlingi*	27			
	*An. nuneztovari*	1			
	*An. triannulatus*	2			
Camp Bernet	*An. aquasalis*	2	7	0.40	10.2
	*An. braziliensis*	10			
	*An. darlingi*	100			
	*An. evansae*	4			
	*An. mediopunctatus*	1			
	*An. oswaldoi*	1			
	*An. triannulatus*	12			
Espérance	*An. braziliensis*	4	4	0.64	2.0
	*An. darlingi*	16			
	*An. nuneztovari*	1			
	*An. triannulatus*	11			
Lotissement Maripa	*An. braziliensis*	6	4	0.32	6.8
	*An. darlingi*	58			
	*An. evansae*	1			
	*An. triannulatus*	6			
Martin village	*An. braziliensis*	9	5	0.35	12.4
	*An. darlingi*	152			
	*An. nuneztovari*	2			
	*An. intermedius*	1			
	*An. triannulatus*	28			
Onozo	*An. darlingi*	14	2	0.23	1.1
	*An. triannulatus*	2			
Savane	*An. nimbus*	1	3	0.60	0.9
	*An. braziliensis*	7			
	*An. darlingi*	4			
Blondin village	*An. braziliensis*	2	4	0.20	35.6
	*An. darlingi*	548			
	*An. nuneztovari*	10			
	*An. triannulatus*	5			
Saut Maripa	*An. darlingi*	133	2	0.25	26.6
	*An. nuneztovari*	2			

**Figure 2:**
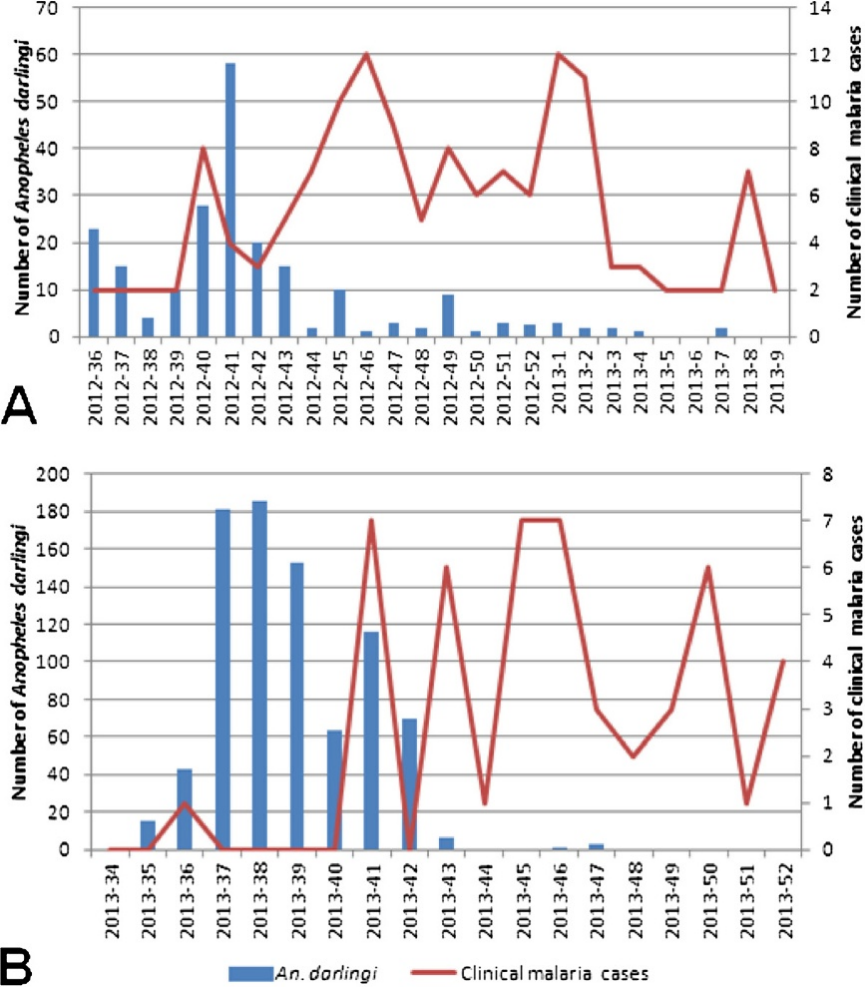
**Evolution in density of** ***Anopheles darlingi*** **and clinical malaria cases recorded from all areas in the study site used for mosquito collection. A** and **B** respectively represent first and second periods of experimentation.

During the two study periods, 192 malaria cases were recorded in the study site. Majority of the cases was due to *P. vivax* (93.3%, n = 179) while *Plasmodium falciparum* accounted for 6.7% (n = 13). Five patients had a second episode of malaria caused by *P. vivax* and was considered as new infections. Dynamics in *An. darlingi* densities and malaria cases observed during this study are presented in Figure 2. For both collection periods, increase in *An. darlingi* density was followed by increase in malaria cases. During the first period, density of *An. darlingi* was highest in week 41 of 2012 and malaria cases were highest in week 45 of 2012 and week 1 of 2013 (Figure 2A). During the second period, density of *An. darlingi* was highest in week 38 of 2013 and malaria cases were highest in week 41 of 2013 and between weeks 45 and 46 (Figure 2B).

**Figure 3:**
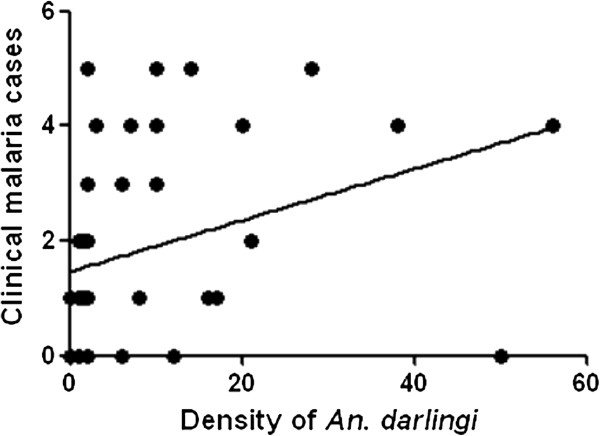
**Linear regression analysis of density of adult female** ***An. darlingi*** **obtained using MMoct from five areas maintained during the two study periods and six weeks lag clinical malaria cases.**

Correlations between *An. darlingi* densities observed each week and the number of weekly clinical malaria cases diagnosed (all *Plasmodium* species together), was investigated for both study periods using Spearman test (Figure 3). Results indicated a significant and positive correlation (p = 0.0001; r = 0.5748) with a six weeks lag time in-between the two variables.

## Discussion

Monitoring of malaria vectors forms an integral part of any malaria control program and this relies on effective sampling. Currently, the reference sampling method for malaria vectors is HLC, which continues to suffer from intense criticism [23, 24]. Over the past decades, multiplicities of proposed alternative sampling methods have been developed. In French Guiana, limited studies have compared these available alternatives so as to replace HLC as a sampling method for anopheline mosquitoes [15]. The present study was, therefore, designed to: (i) determine an alternative trapping system that can replace HLC for follow-up of Amazonian anopheline mosquitoes, particularly *An. darlingi,* and (ii) assessing the possible use of MMoct in collecting entomological data that can assist in predicting emergence of malaria.

Mosquito Magnet trap fitted with either octenol or Lurex™ lures was evaluated against HLC for sampling anopheline mosquitoes. MMoct have been shown to be more efficient than CDC light trap for surveillance of mosquitoes [15, 25, 26]. The only such study in French Guiana used MMoct [15] and hence MMlur was included in the current study. Data obtained from this study clearly shows that in terms of density and diversity, HLC remains a better method for sampling anopheline mosquitoes in French Guiana. It was only seconded by MMoct, which showed high species diversity. The observed performance of HLC tallies with findings by other authors who found HLC to be an appropriate method for sampling anthropophilic anopheline mosquitoes [25, 27, 28]. The huge difference in mosquito density estimated using HLC and MM can be explained by the fact that, humans produce many cues for attracting mosquitoes than MM. By the current study, the number of mosquitoes collected by MMoct needs to be multiplied by 20 to extrapolate collection by HLC. For a trapping method to be relevant, it is of paramount importance that it does not only collect mosquitoes in large number. It should also significantly collect the main malaria vectors and most importantly, population of the vector at risk of transmitting malaria. In the context of the Amazonian basin and specifically French Guiana, where *An. darlingi* is the main malaria vector [4, 5], MMoct and MMlur performed well in collecting this species even though MMoct was slightly better. Parity rate, an entomological indicator used to estimate the age of insect population was investigated. The 52.3% parity rate obtained in the current study represents the proportion of *An. darlingi* females that have taken blood meal and laid eggs at least once and this older population allows for increase feeding which increases the chance of the mosquitoes getting infected or infecting uninfected humans. Interestingly, MMoct significantly collected more parous adult female *An. darlingi* mosquitoes than the other sampling methods which in a way can decrease the population of potentially infective mosquito. However, this should be interpreted with caution since the sample size dissected for parity was not equal for each sampling method. In addition to *An. darlingi*, both HLC and MMoct collected *An. triannulatus* and *An. nuneztovari*. These two species are known vectors of malaria in other South American countries [29, 30]. However, their role in malaria transmission in French Guiana is still unclear. In this study, MMoct out performed HLC in sampling *An. triannulatus* and this is in line with the observations made by Rubio-Palis *et al*. [31]. The low numbers of *An. triannulatus* collected by HLC, might suggest zoophilic behaviour of this species in French Guiana. Results from this investigation imply that MMoct had similar efficiency with HLC in sampling *An. nuneztovari*, showing its potential to replace HLC in French Guiana. The main advantages of MM trap lies in its functional longevity. It can function for weeks without needing additional propane. Lures can be switched in a single MM trap and also the fact that the equipment is user friendly. However, continuous recharging of battery and difficulties associated in transporting heavy gas bottles in the field suggest that the technology needs to be improved.

A significant proportion of unidentified mosquitoes were collected using MM traps during the longitudinal study. Mosquitoes caught using this trap where often left for longer period (four days) in the traps and recovered dried with damage to morphological characters, making it difficult to identify. This was not the case with mosquitoes that were recovered from traps within 24 hours, as was done during one week collection by the authors each month. The mosquitoes were usually fresh and undamaged, hence were easily identified and dissected for parity. While mosquitoes can frequently be recovered from the trap, damage to morphologically diagnostic features of specimens, highlights the need for a molecular base identification technique such as PCR.

Varying species richness and diversity index values were calculated for collection methods and collection areas. HLC had the highest species richness and MMoct the highest species diversity index. This differs from results obtained by Dusfour *et al.* [15] who found the reverse to be true. Both studies were conducted in inland primary forest with similar but yet different ecological settings. This is reflected in the differences in anopheline species composition in both study and most likely explains the contradictory results. Similar relationship between species richness and diversity index as seen between collection methods in Blondin village, was also recorded in areas around the town of Saint-Georges. The highest species richness was found in Camp Bernet while the highest species diversity index was obtained in Blondin village. The low Simpson diversity index experienced in Camp Bernet can be explained by the unevenness in the densities of species collected from this area. In French Guiana where other anopheline species have been found naturally infected with *Plasmodium* parasite and suspected to play a role in malaria transmission [6], a trap able to catch a wide range of anopheline mosquitoes is relevant to malaria vector control programs. Knowing the species present will help in the design and implementation of malaria vector control strategies. It will be interesting to know how the richness and evenness of these species influences malaria transmission in the town of Saint-Georges. High densities of anopheline mosquitoes were collected from Blondin village, Saut Maripa and Martin, characterized by forest environment and located close to the Oyapock River. The forest areas are usually flooded by water from the river and its tributaries during tidal effects, creating slow flow forest pools which may provide suitable breeding ground for *An. darlingi*.

Malaria transmission is best assessed by the entomological inoculation rate (EIR), and is commonly calculated as a product of vector density or vector biting rate and sporozoites rate [32]. The current study also aimed at measuring malaria transmission, using entomological indicators obtained from mosquitoes collected with MMoct traps and to validate with epidemiological data. Unfortunate, this was not done because none of the mosquitoes were infected with *Plasmodium* sporozoites. The lack of infected mosquitoes can be a consequence of various reasons. Firstly, the sample size screened was low. Secondly, there is a possibility that the traps were located in areas with no malaria transmission. In fact, it is well-known that malaria transmission can differ significantly within localities in a village located even a mile from each other [10]. Lastly, infected mosquitoes could in fact have been repelled by chemicals in the lure, a hypothesis that remain to be proved scientifically. No matter what the exact reason is, not finding an infected mosquito is worrying because collection areas were chosen based on epidemiological information and traps were located close to people’s homes. Also, not enough specimens of *An. darlingi* were available to be dissected for parity when collections were done during the four days of the first week of each month. Specimens collected by the contracted association were stored for at least a month and were usually too dry to be dissected. Consequently, the longevity of *An. darlingi* could not be estimated for analysis with malaria cases. However, the density of *An. darlingi* in this study significantly correlated with clinical malaria cases and again, highlighting the potential of MMoct for surveillance of malaria vectors in French Guiana.

## Conclusion

Efficient sampling of malaria vector species is critical for any malaria vector control programme. Sampling systems should be chosen most importantly in the context of the main vector responsible for malaria transmission. In the current study, MMoct and MMlur did not drastically differ in their efficacy for sampling the main malaria vector *An. darlingi*. However, MMoct emerged the most appropriate trapping system based on its ability to collect more anopheline species. MMoct seems promising in providing reliable entomological information that can facilitate the development of early warning systems for malaria surveillance.
